# The Prevalence and Impact of Migraine Among Physicians in Saudi Arabia: A Cross-Sectional Study

**DOI:** 10.7759/cureus.70607

**Published:** 2024-10-01

**Authors:** Rami Algahtani, Renad Melebary, Lujain A Alzahrani, Aseel M Alahmadi, Nada Almalayo, Razan Melebary

**Affiliations:** 1 Medicine, Umm Al-Qura University, Makkah, SAU; 2 Medicine, Mecca Health Cluster, Makkah, SAU; 3 Pediatrics, King Abdulaziz Medical City, Jeddah, SAU

**Keywords:** doctors, headache disorders, migraine, prevalence, quality of life

## Abstract

Background

Migraine is a primary headache disorder and one of the most common causes of disability worldwide. Prior reports showed a higher prevalence of migraine among physicians. We conducted this study to estimate the prevalence of migraine among physicians in Saudi Arabia, as well as its impact on quality of life.

Methods

A cross-sectional study was conducted using a validated online questionnaire that was distributed among physicians in various medical and surgical specialties in Saudi Arabia from January to June 2023. The inclusion criteria involved physicians in all specialties, who worked in governmental or private hospitals in Saudi Arabia. Employed or healthcare workers other than physicians were excluded. To measure the impact of headache on a person’s quality of life, the Migraine Disability Assessment (MIDAS) test was applied. Statistical analysis of the study was conducted using the Statistical Package for the Social Sciences (SPSS) Version 24 (IBM Corp., Armonk, NY).

Results

We had 387 respondents, of whom 362 fulfilled the inclusion criteria. The mean age was 33.02 ± 9.07 years, and genders were distributed equally. Most physicians worked in departments of internal medicine, family medicine, general practice, and general surgery.

The prevalence of migraine was recorded in 17.1% (62 patients) of all the included participants, with a female predominance. MIDAS revealed a total mean score of 16.823±14.149. The severity grades were classified as moderate (37.1%), severe (35.5%), or little to no (21.0%). Stress and light were cited as the top factors that trigger or worsen migraine, while sleep, rest, and quiet and darknesswere cited as the top relieving factors. The average number of headache attacks (month) was 6.823. Most patients with migraine were board residents (27.4%), living in the western region (53.2%), and working in a governmental hospital (38.7%).

Conclusion

Migraine is a common disorder among physicians in Saudi Arabia, with significant moderate-to-severe disability. Stress is identified as a major trigger. Proper awareness, stress management programs, and therapeutic intervention may mitigate the untoward consequences of migraine.

## Introduction

Migraine disorder is one of the most common causes of disability, affecting nearly 12% of the general population, with females being more likely to be affected than males [1]. A higher prevalence of migraine has been noted among healthcare workers [2], largely attributed to the stressful nature of their work, as work-related stress is considered a risk factor for migraine [3]. This inevitably impacts their work productivity and the quality of their performance [4,5].

Migraine is a genetically influenced disorder characterized by episodes of unilateral pulsating headaches that last up to 72 hours and are frequently accompanied by nausea, as well as sensitivity to light and sound [1]. The International Headache Society (IHS) has classified migraine into many subtypes, with the most common being migraine without aura [6]. Various triggers have been reported to play a role in the development of migraine attacks, with the following triggers being present in more than 50% of migraine patients: stress hormone changes, skipped meals, weather changes, sleep inadequacy, or oversleeping [1]. According to the Global Burden of Diseases study, migraine is one of the leading causes of years lost due to a disabling medical condition worldwide [7]. The prevalence of anxiety and depression among people with migraine is significantly higher as a result of suffering from a chronic disorder. Due to its chronic course, migraine can significantly impair quality of life. Patients usually report a poorer quality of life compared to healthy individuals, particularly in aspects of emotional and physical well-being [8]. There have been few recent studies estimating the number of migraine patients in Saudi Arabia. According to a cross-sectional study, 1,333 (26.77%) of 4,943 participants had migraine [9].

Migraine can interfere with many personal and social needs and activities. Work productivity is an important aspect of an individual’s life that can be directly affected by migraine. Presenteeism is defined as a loss of work productivity due to a particular health condition [4], which is common among patients with migraine. A systematic review conducted in the United States showed that two to three workdays per month were lost due to migraine [10]. Another study from the Philippines demonstrated that over three months, nine days would be affected by migraine [11]. All those lost days can result in defects in work systems. Among many stressful work environments, physicians consistently work in a particularly stressful one. It is known that stress is a common trigger of migraine, believed to be due to the activation of the hypothalamic-pituitary-adrenocortical axis and the sympathetic nervous system [12]. Therefore, physicians are more likely to experience migraine attacks, which may have numerous consequences on their quality of life, work productivity, healthcare quality, and the healthcare system's economy. Our study aims to estimate the prevalence of migraine among physicians and its impact on their quality of life in Saudi Arabia.

## Materials and methods

A cross-sectional study design was conducted using an online self-administered questionnaire to collect data from physicians of different specialties in Saudi Arabia. The data collection period started from January 2023 up till June 2023. The sample size was calculated to be 384, using the single proportion equation in OpenEpi Calculator website [13], at 95% confidence intervals and a 5% accepted margin of error. However, the study sample was increased to 400 to compensate for any dropout. A simple random sampling technique was applied. The total sample (n=400) was proportionally distributed among physicians. Regarding the inclusion criteria, the study included doctors in all specialties, who work in governmental or private hospitals in Saudi Arabia, regardless of their positions (consultants, specialists, residents, service), age, gender, and nationality, and who agreed to participate in this study. Participants who did not give consent or were unable to participate in our study, as well as employees other than doctors, were excluded. A self-administered questionnaire on Google Forms was used for electronic data collection. Various methods were used to contact the physicians, such as different social media apps, phone calls, and direct communication in the hospitals. Consent was obtained at the beginning of administering the questionnaire, and all data were kept confidential. The questionnaire consisted of three sections: demographic data, diagnostic criteria of migraine headache and its associated factors, and quality of life assessment via the Migraine Disability Assessment (MIDAS) test (Appendix). To measure the impact of headache on a person’s quality of life, the valid and reliable MIDAS [14] test was applied. A pilot study was conducted on 10 participants, whose responses were not included in the main study. The purpose of the pilot study was to assess the validity and reliability of the study questionnaire and to identify the necessary modifications that were carried out accordingly. The Statistical Package for Social Sciences (SPSS)Version 25.0 (IBM Corp., Armonk, NY) was used for data entry and analysis.

Statical analysis

Statistical analysis of the study was conducted using SPSS Version 24, while figures were displayed using RStudio (R version 4.3.1, R Foundation for Statistical Computing, Vienna, Austria). Frequencies and percentages were used to express the categorical variables, while numeric data were presented as mean ± SD. Items with multiple responses were analyzed using the multiple-response analysis technique. The prevalence of migraine was determined based on either applying the diagnostic criteria of episodic migraine headache of the International Headache Society (IHS) [6] or via a direct question of previous professional diagnosis by a physician in neurology. To determine the quality of life, the MIDAS test was applied [14]. Hence, the score consisted of the following four severity grades that predict the patient’s treatment needs: grade I (score of 0-5) indicates little or no disability, grade II (score of 6-10) indicates mild disability; grade III (score of 11-20) indicates moderate disability, and grade IV (score of ≥ 21) indicates severe disability. Factors associated with migraine headaches were assessed using Pearson’s chi-squared test or Fisher’s exact test whenever applicable. A p-value of <0.05 indicated statistical significance.

## Results

Sociodemographic characteristics

**Figure 1:**
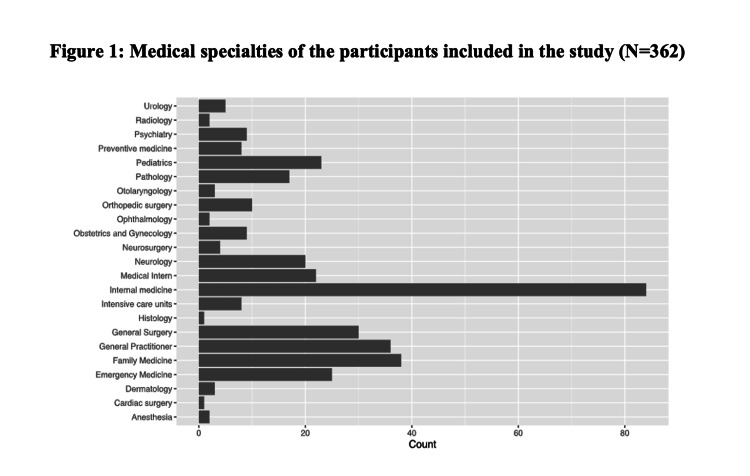
Medical specialties of the participants included in the study (N=362).

A total of 362 physicians were included in the study. Hence, the gender showed an equal distribution between males and females (181, 50.0% for each group). The mean age was 33.02 ± 9.07 years, and the most common age group was 25-34 years (63.5%), followed by 35-44 years (20.4%) and 45-54 years (7.2%). Concerning the medical career profile, most of the participants were board residents, general practitioners, and consultants (31.8%, 22.9%, and 18.0%, respectively). Nearly half of the included physicians worked in governmental hospitals (177, 48.9%), while the least of participants worked in private hospitals (16, 4.4%). Additional data concerning demographic and work-related information are represented in Table 1. The medical specialties of the participants were obtained and are demonstrated in Figure 1. Hence, most of the physicians worked in the departments of internal medicine, family medicine, general practice, and general surgery. The least specialties reported were cardiac surgery, histology, ophthalmology, and anesthesia.

**Table 1:** Demographic characteristics of the participants (N=362).

Variable		No	%
Age (mean ± SD)		33.02 ±9.07	
Years of experience in the medical field (mean ± SD)		6.83 ±7.91	
Age	18-24 years	16	4.4%
	25-34 years	230	63.5%
	35-44 years	74	20.4%
	45-54 years	26	7.2%
	55 years and over	16	4.4%
Gender	Female	181	50.0%
	Male	181	50.0%
Nationality	Saudi	315	87.0%
	Non-Saudi	47	13.0%
Social status	Single	165	45.6%
	Married	181	50.0%
	Divorced or widow/er	16	4.4%
Medical degree	Board resident	115	31.8%
	General practitioner	83	22.9%
	Fellow	17	4.7%
	Specialist	49	13.5%
	Consultant	65	18.0%
	Other medical degree	33	9.1%
Place of work	Governmental hospital	177	48.9%
	Military hospital	32	8.8%
	Private hospital	16	4.4%
	Specialty hospital	48	13.3%
	Primary health care center	27	7.5%
	University (teaching) hospital	62	17.1%
Region of residency	Central region	37	10.2%
	Eastern region	94	26.0%
	Western region	181	50.0%
	Southern region	30	8.3%
	Northern region	20	5.5%

Prevalence of migraine headache and its associated factors

**Figure 2:**
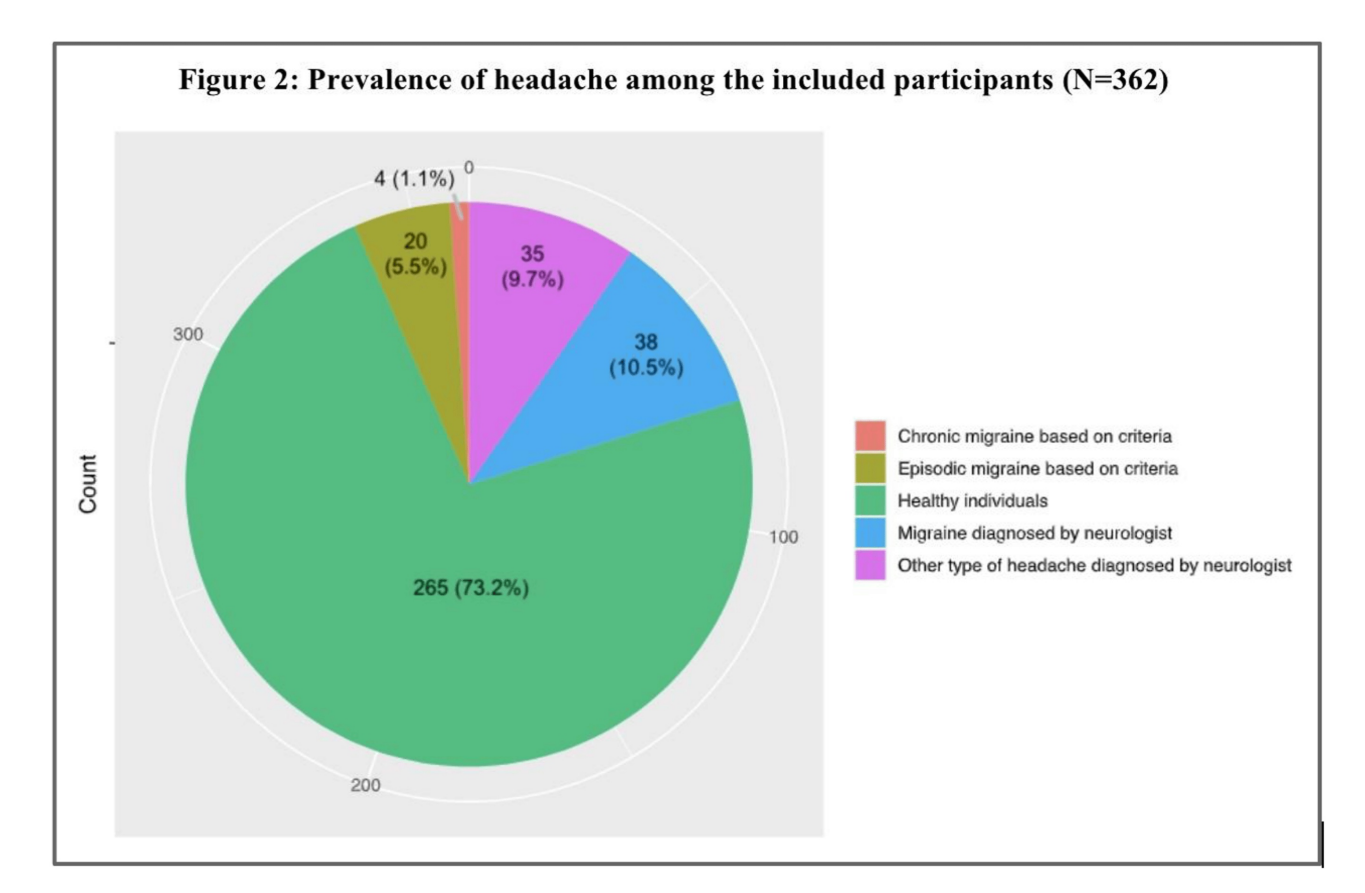
Prevalence of headache among the included participants (N=362).

The prevalence of migraine was recorded among 17.1% (62 patients) of all the included participants. Hence, 38 patients were diagnosed with migraines by a neurologist, while the HIS criteria revealed 20 cases with episodic migraines and four cases with chronic migraines. Additional data concerning headache prevalence are presented in Figure 2. Among those patients, more than half described the migraine attacks as throbbing or pulsating in nature (71.0%), unilateral (80.6%), and aggravated by physical activity (62.9%). Additional factors associated with migraine headaches are shown in Table 2.

**Table 2:** Assessment of factors associated with migraine headache among migraine patients (N=62). ±A specific question for all individuals who participated in the study. ѯMultiple-choice question; hence, the percentage represents the frequency of each symptom rather than the whole population. £Such as menstrual period, pregnancy, use of oral contraceptive pills, and use of other hormonal drugs.

Variables		No	%
Number of headache attacks (month) (mean ± SD)		6.823 (5.168)	
Smoking status±	Current smoker	73	20.2%
	Non-smoker/ex-smoker	289	79.8%
Headache duration if left untreated (hours)	4-12 hours	37	59.7%
	13-24 hours	17	27.4%
	>24 hours	8	12.9%
Headache description	Throbbing or pulsating	44	71.0%
	Pressing, squeezing, or tightening	18	29.0%
Site of headache	Unilateral	50	80.6%
	Bilateral	12	19.4%
Physical activity (e.g., walking or climbing stairs)	Headache aggravated by physical activity	39	62.9%
	Headache is not aggravated by physical activity	23	37.1%
Concomitant symptoms	Nausea	45	72.6%
	Vomiting	15	24.2%
	Photophobia	52	83.9%
	phonophobia	55	88.7%
Aura symptomsѯ	Blurred/double/loss vision or zigzag lines appear	23	37.1%
	Numbness or tingling	11	17.7%
	One side of the body weakness	9	14.5%
	Speech difficulty	5	8.1%
	None	33	53.2%
Factors that bring on headaches or make them worseѯ	Stress	54	87.1%
	Hormones in women£	24	38.7%
	Missed meals	27	43.5%
	Weather change	18	29.0%
	Sleep disturbance	0	0.0%
	Certain smells or perfumes	15	24.2%
	Lights	35	56.5%
	Smoking	12	19.4%
	Certain foods, exercise, or sexual activity	8	12.9%
	None of the above	1	1.6%
Factors that make headaches better/relievingѯ	Rest	45	72.6%
	Sleep	43	69.4%
	Quiet and darkness	44	71.0%
	Exercise	1	1.6%
	Massage	2	3.2%
	Warm shower	14	22.6%
	None of the above	2	3.2%
Medication historyѯ	Analgesic	24	38.7%
	NSAIDs	23	37.1%
	Amigraine	2	3.2%
	Botox injection	2	3.2%
	Triptan/Sumatriptan	7	11.3%
	Beta-blocker	7	11.3%
	Amitriptyline	5	8.1%
	Riboflavin	1	1.6%
	Caffeine	3	4.8%
	Not on any medication	21	33.9%
Family history of migraineѯ	Mother	18	29.5%
	Father	3	4.9%
	Siblings	20	32.8%
	Second-degree relatives	7	11.5%
	No known family history of migraine	24	38.7%
Past medical historyѯ	Hypertension	5	8.06%
	Diabetes mellitus	2	3.23%
	Hypothyroidism	1	1.61%
	Asthma	12	19.35%
	Peptic ulcer	1	1.61%
	No known past medical history	40	64.5%
	Others	2	3.23%

Quality-of-life assessment via MIDAS

**Figure 3:**
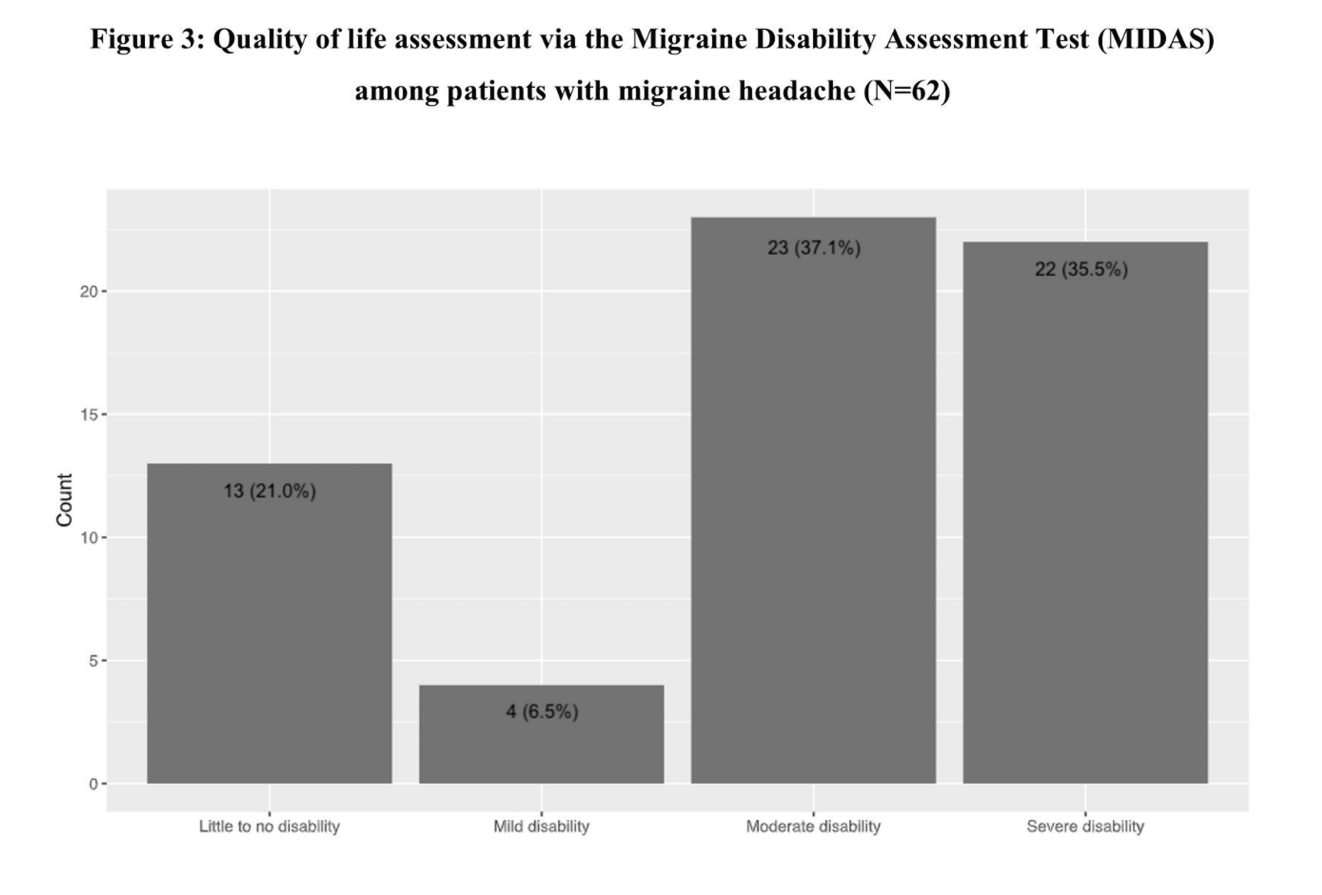
Quality-of-life assessment via the MIDAS test among patients with migraine headache (N=62) MIDAS, Migraine Disability Assessment

The MIDAS test revealed a total mean score of 16.823±14.149. Moreover, the distribution of severity grades among migraine patients shows nearly equal percentages of moderate and severe disability (37.1% and 35.5%, respectively), followed by little to no disability (21.0%); only four patients recorded mild disability (6.5%) (Figure 3).

Factors associated with migraine headache

Data regarding factors associated with migraines are given in Table 3. Higher mean scores of MIDAS were associated with a statistically significant recorded p-value (< 0.001). Furthermore, migraine patients were commonly females (71.0%) rather than males (45.7%), with a significant association recorded with a p-value of <0.001. Most patients with migraine were board residents (27.4%), living in the western region (53.2%), and working in a governmental hospital (38.7%). A negative family history of migraine and little or no disability on MIDAS score were associated with the non-migraine group, with a statistically significant p-value (<0.001).

**Table 3:** The association between migraine and other factors. *As p-value is <0.05, it represents statistical significance

Variables		Migraine group (N=62)		Non-migraine (N=300)		p-Value
		Freq	%	Freq	%	
Years of experience	Mean (SD)	5.790 (6.869)		7.047 (8.102)		0.255
	Median (Q1, Q3)	4.0 (1.250, 7.0)		4.0 (1.0, 10.0)		
Total MIDAS score	Mean (SD)	16.823 (14.149)		5.860 (10.506)		<0.001*
	Median (Q1, Q3)	16.0 (6.3, 22.8)		1.0 (0.0, 7.0)		
Age group	18-24 years	7	11.3%	9	3%	0.068
	25-34 years	37	59.7%	193	64.3%	
	35-44 years	11	17.7%	63	21.0%	
	45-54 years	5	8.1%	21	7.0%	
	55 years and over	2	3.2%	14	4.7%	
Gender	Female	44	71.0%	137	45.7%	<0.001*
	Male	18	29.0%	163	54.3%	
Nationality	Saudi	57	91.9%	258	86.0%	0.206
	Non-Saudi	5	8.1%	42	14.0%	
Marital status	Married	28	45.2%	153	51.0%	0.267
	Single	33	53.2%	132	44.0%	
	Divorced/Widowed	1	1.6%	15	5.0%	
Medical degree	Board resident	17	27.4%	98	32.7%	0.067
	General practitioner	11	17.7%	72	24.0%	
	Fellow	2	3.2%	15	5.0%	
	Specialist	9	14.5%	40	13.3%	
	Consultant	11	17.7%	54	18.0%	
	Other medical degree	12	19.4%	21	7.0%	
Smoking status	Current smoker	12	19.4%	51	20.39%	0.861
	Non-smoker/ex-smoker	50	80.6%	239	79.7%	
Region of residency	Central region	2	3.2%	35	11.7%	0.389
	Eastern region	18	9.0%	76	25.3%	
	Western region	33	53.2%	148	49.3%	
	Southern region	5	8.1%	25	8.3%	
	Northern region	4	6.5%	16	5.3%	
Place of work	Government hospital	24	38.7%	153	51.0%	0.044
	Military hospital	6	9.7%	26	8.7%	
	Private hospital	6	9.7%	10	3.3%	
	Specialty hospital	8	12.9%	40	13.3%	
	Primary healthcare	2	3.2%	25	8.3%	
	University hospital	16	25.8%	46	15.3%	
Family history of Migraine	Positive history	38	61.3%	81	27.0%	<0.001*
	Negative history	24	38.7%	219	73.0%	
MIDAS disability	Little to no disability	13	21.0%	213	71.0%	<0.001*
	Mild disability	4	6.5%	32	10.7%	
	Moderate disability	23	37.1%	31	10.3%	
	Severe disability	22	35.5%	24	8.0%	

## Discussion

Migraine, tension-type, and cluster headache are all considered primary headache disorders. Due to their high prevalence and significant impact on the quality of life, they represent a main global health problem that should not be underestimated. This study illustrates the prevalence of migraine among physicians in Saudi Arabia. In our sampled population, the prevalence of migraine was 17.1%. This is higher than the prevalence reported by Al-Qurashi and Alsaedi, which was 12.7% among primary healthcare workers in Makkah, Kingdom of Saudi Arabia [15]. However, a higher prevalence was noted in another previous study conducted in Abha city, which showed a prevalence of 20.8% among primary healthcare workers [2]. In addition to the prevalence, when compared with the study, we found that the mean number of attacks, which was 6.8 per month, was much higher than the mean (four attacks) reported by Al-Qurashi and Alsaedi [15]. These variations could be attributed to several socioeconomic, regional, and populational characteristics including participants in different cities and medical specialties.

Our findings are consistent with those of several research studies. According to a systematic review that was published in 2020, migraine prevalence in Arab nations varied from 2.6% to 32%, with the Kingdom of Saudi Arabia reporting the highest prevalence. The rates among medical students varied from 12.2% to 27.9%, ranging from 7.1% to 13.7% among schoolchildren [3]. These findings suggest that a person's job or position may have an impact on the prevalence of migraines. According to research by Abdul Jabbar and Ogunniyi including 5,891 members of the general population, professionals were more likely to get migraines, presumably as a result of high levels of stress [16].

More than half of the physicians included in this study described their headaches as pulsatile, unilateral, and throbbing in nature. This is similar to the findings of the study conducted in Abha [2]. Photophobia and phonophobia were the most frequently encountered symptoms.

When assessing the severity grades within our study's cohort of migraine patients using the MIDAS test, a distinct pattern emerges. A significant proportion (37.1%) of patients reported a moderate level of disability, closely followed by severe disability (35.5%). In contrast, a minority of patients (21.0%) reported little to no disability. Notably, these findings deviate from those reported by Al-Qurashi and Alsaedi, whose research suggests a distribution of 40.0% for little or no disability, followed by 35.0% for moderate disability [15]. However, according to the study conducted in Al-Ahsa region of Saudi Arabia, over half of migraine patients (57.3%) experienced severe disability [17].

A minimal or no disability, as indicated by the MIDAS score and negative family history, demonstrated a significant association with the non-migraine group. These findings could collectively underscore the proposition that genetic factors may contribute to the manifestation of migraine.

Regarding the factors associated with migraines, our study reveals a higher prevalence among females (71.0%) compared to males (45.7%). This observation aligns with numerous prior studies that consistently report a greater susceptibility of females to migraines compared to males for a variety of genetic and hormonal differences [18,19].

Moreover, a significant outcome in the current study is the preponderance of board residents within the patient cohort, accounting for 27.4%. This finding resonates with a previously published study conducted in Abha [18]. Additionally, our results unveil that a substantial proportion of physicians (38.7%) were employed in governmental hospitals. This observation may be attributed to the heightened workload typically experienced in governmental hospitals, offering insight into the occupational distribution of migraine cases within our study population.

Our study is limited by its cross-sectional nature, which precludes tracking changes over time. The study was conducted via an online questionnaire, which expose the study to the risk of social-desirability bias and makes it difficult to apply inclusion and exclusion criteria stringently.

## Conclusions

This study emphasizes the high prevalence and significant impact of migraines among physicians in Saudi Arabia. The findings contribute to the growing body of evidence on the global burden of migraines and the need for increased awareness, prevention, and management strategies in healthcare settings. There is a need for further research investigating the specific factors contributing to migraines among physicians, as well as interventions to mitigate the impact of migraines on their well-being and healthcare consistency. By addressing this issue, healthcare system can better support physicians in managing migraines and enhancing their overall quality of life.

## Appendices

The online self-administered questionnaire.

Section 1: Demographic Factors

1. Age (years):

-18-29

-30-39

-40-49

-50-59

-60+

2.Nationality:

-Saudi

-Non-Saudi

3. Gender:

-Female

-Male

4. Where do you live (city)?

5. Where do you work?

-MOH general hospitals

-Specialty hospital

-University(teaching) hospital

-Military hospital

-Primary healthcare center

-Private hospital

-Other

6. What is your specialty?

7. Are you a…?

-General practitioner

-Board junior resident

-Board senior resident

-Specialist.

-Fellow.

-Consultant.

-Other, mention:

8. Do you smoke?

-Yes

-No

9. Do you have any chronic diseases?

-Cardiovascular diseases

-Neurological diseases

-Hypertension

-Diabetes

-Obesity

-Thyroid disorders

-Depression

-Anxiety

-Others

-None

Section 2: Migraine-Related Questions

1-Are you diagnosed with migraine?

-Yes

-No

2. If yes, for how long have you been diagnosed with migraine?

-Less than 12 months

-1-2 years

-3-5 years

-More than 5 years

3. Number of migraine attack per month:

-1-2

-3-4

-5-6.

-More than 7

 Section 3: Quality-of-Life Assessment via The Migraine Disability Assessment (MIDAS) test

Please answer the following questions about ALL of the headaches you have had over the last 3 months. Select your answer in the box next to each question. Select zero if you did not have the activity in the last three months. Please take the completed form to your healthcare professional.

1. On how many days in the last three months did you miss work or school because of your headaches?

2. How many days in the last three months was your productivity at work or school reduced by half or more because of your headaches? (Do not include days you counted in question 1 where you missed work or school.)

3. On how many days in the last three months did you not do household work (such as housework, home repair and maintenance, shopping, caring for children and relatives) because of your headaches?

4. How many days in the last three months was your productivity in household work reduced by half or more because of your headaches? (Do not include days you counted in question 3 where you did not do household work.)

5. On how many days in the last three months did you miss family, social, or leisure activities because of your headaches?
